# Genomics for clinical utility: the future is near

**DOI:** 10.1186/gm522

**Published:** 2014-01-28

**Authors:** David Rossolatos, Katherine J Aitchison

**Affiliations:** 1 Department of Psychiatry, University of Alberta, Edmonton, AB T6G 2E1, Canada; 2 Department of Medical Genetics, University of Alberta, Edmonton, AB T6G 2E1, Canada

## Abstract

A report on the Precision Medicine: Personal Genomes and Pharmacogenomics meeting, Cold Spring Harbor Laboratory, USA, November 13–16, 2013.

## Bench to bedside

Precision medicine has come a long way in the past year. Whole exome sequencing is now being used clinically, with the exponential growth in the number of exomes sequenced in recent years resulting in data repositories that aim to facilitate clinical interpretation. This wealth of information is prompting researchers and clinicians to question their responses to secondary findings and to ask who owns this valuable genetic information. Understanding these data will require developments in bioinformatics and, crucially, databases, and the scale of recent advances has led to an exploration of cloud computing technologies. The rate of progress is now so fast that Richard Durbin (Wellcome Trust Sanger Institute, UK) made the prediction that it is conceivable we will reach the point where every genomic variation possible will have been observed.

Of all the applications of genomics to precision medicine, genomic diagnostics stands out for its remarkable progress, and this was the theme of the first session of the meeting. Hugh Rienhoff (Children’s Hospital Oakland Research Institute, USA) opened the meeting by describing his journey to find a genomic answer to his daughter’s condition, and the eventual identification of a functional mutation in the gene for transforming growth factor-beta. A larger scale search for genomic answers was outlined by Leslie Biesecker (National Human Genome Research Institute, National Institutes of Health, USA), whose team has developed an integrated genome-to-phenotype approach to assess the clinical consequences of a set of loss-of-function variants in a cohort of 870 exomes, a hypothesis-generating approach described as customized phenotyping. A clinically significant phenotype was found in approximately 2% to 3% of patients, which was higher than expected.

About half of current attendees at medical genetic clinics do not have a molecular diagnosis. Christine Eng (Baylor College of Medicine, USA) presented the Baylor pipeline for whole exome sequencing in a clinically accredited laboratory to identify causative mutations underlying disease phenotypes; this approach was successful in approximately 25% of undiagnosed patients. The Baylor team filter their results with a regularly updated, internally annotated gene list before clinical reporting by a team of clinicians and genetic counselors; they also implement three levels of review, ending up with a ‘sign-out’ conference. They report the 56 medically actionable genes recommended by the American College of Medical Genetics, but also include mutations that are predicted to be deleterious in genes that do not currently have an association with disease. Of note, 11 patients (of the 250 examined) had two molecular diagnoses, challenging the traditional aim to find a ‘single unifying diagnosis’.

Tim Hubbard (Wellcome Trust Sanger Institute, UK) outlined the 100 K Genome Project (Genomics England), which aims to sequence up to 100,000 patients over the next five years to identify pathogenic mutations for rare diseases, common cancers with high mortality, and infectious diseases. Companies able to provide annotation that can run inside the National Health Service firewall as an app will be sought in a competitive process. Patients will receive their data if they consent to do so.

Ekta Khurana and Michael Cromer (Yale University, USA) presented work from the functional group of the 1000 Genomes project, in which non-coding candidate cancer driver mutations were identified in somatic variants. A specific example was a somatic mutation that alters the DNA-binding motif of transcription factor YY1 found in 14 patients with insulin-producing adenomas. Subsequent exome sequencing identified that one-third of insulinomas had this mutation, which was not found in other types of pancreatic tumors.

Genomics is also being applied to understand the progression of disease and especially to uncover the mechanisms of acquired resistance. Maja Krajinovic (Centre Hospitalier Universitaire Sainte-Justine Research Center, Canada) described her study of the 20% of patients with acute lymphoblastic leukemia who are resistant to treatment with asparaginase (a standard component in the treatment of childhood acute lymphoblastic leukemia). She identified mutations in *ATF5* and *ASNS* associated with an adverse drug reaction to asparaginase derived from *Escherichia coli*. Also in this session, Christopher Miller (Washington University School of Medicine, USA) described methods for detecting cryptic subclones in cancer that might be resistant to treatment and result in post-treatment metastasis.

## Looking for the limit in locus heterogeneity

One of the most successful applications for exome sequencing and related techniques to date has been the detection of copy number variants. Mathew Hurles (Wellcome Trust Sanger Institute, UK) provided an update on the Deciphering Developmental Disorders initiative, which has so far recruited 7,048 families out of the planned 12,000 families. By using exome arraycomparative genomic hybridization, the consortium has shown that 12% of children have *de novo* single nucleotide variants or indels. Of note, 2.7% of children previously assessed with a clinical grade comparative genomic hybridization microarray were found to have pathogenic copy number variants, compared with 6.1% of those with no previous clinical grade microarray. Claudia Gonzaga-Jauregui (Baylor College of Medicine, USA) demonstrated the power of exome sequencing and custom array comparative genomic hybridization in identifying genes causative for Charcot-Marie-Tooth disease, for which the symptom complex includes neuropathy. Over 40% of a group of 40 patients with a neuropathy without a known genetic cause were provided with a genetic diagnosis and, interestingly, many patients had mutations in more than one neuropathy gene. Tomasz Gambin (Baylor College of Medicine, USA) presented 2,211 exomes from the Baylor dataset and 232 exomes from Johns Hopkins University, USA, with many unique variants identified (more than 50% novel). There was some difference in pathological classification using the Human Gene Mutation Database in comparison with the ClinVar database (National Center for Biotechnology Information, USA), although the results were broadly similar.

The session on genomic variation in common traits and disease drew a lot of attention. One highlight was the presentation by Lauren Weiss (University of California Santa Cruz, USA), who reported a systematic evaluation of autism traits in the RASopathies caused by activating mutations in the Ras/MAPK signaling pathway: neurofibromatosis type 1, Noonan syndrome, Costello syndrome and cardiofaciocutaneous syndrome. Probands with the four major RASopathies were compared to individuals with idiopathic autism spectrum disorders and siblings with no disorders (controls); those with RASopathies were shown to have more severe autism traits. Genome-wide association study data provided evidence of gene-gene interactions between the Ras/MAPK pathway and other genomic loci (similar to those seen in cancer epistasis), resulting in different phenotypes of autism. It was hypothesized that pharmaceuticals developed to target the Ras/MAPK pathway could be useful in developmental disorders. Treatment of these disorders therefore stands to benefit in the short-term from advances in genomic technology applied to personalized medicine.

## Pharmacogenetics in the clinic

The adoption of pharmacogenetics into clinical practice has historically been slow, but it was clear at this meeting that implementation of pharmacogenomics is now underway. Adam Gordon (University of Washington, USA) presented a new high-throughput sequencing platform, PGRNseq, targeting 84 genes (including 2 kb upstream) with strong associations to drug phenotypes. This platform is being deployed by the Electronic Medical Records and Genomics network, in which patients due to receive a relevant drug will be sequenced, actionable variants identified, and this information deposited in the patient’s electronic medical record, with decision support for clinicians. Initial data from the first 900 samples revealed many novel and rare variants, including newly described truncating variants in important pharmacogenes such as *CYP2C19* and *DPYD*.

Mary Relling (St Jude Children’s Research Hospital, USA) outlined the work of the Clinical Pharmacogenetics Implementation Consortium in developing peer-reviewed, publicly available gene-drug pair guidelines that provide specific recommendations on how to use genetic test results to guide prescribing. St Jude uses the DMETPlus Array (an array designed to assay for over 1,900 variants in genes relevant to the absorption, distribution, metabolism and excretion of prescribed medications), along with a *CYP2D6* copy number assay. It has taken 2.5 years to incorporate data from four genes (*CYP2D6*, *CYP2C19*, *TPMT*, *SLCO1B1*) into the electronic medical records. On a related theme, Catherine Brownstein (Boston Children’s Hospital, USA) described the hospital’s Clinical Pharmacogenomics Service, and demonstrated that they have made cost savings following the analysis of a relatively small number of gene-drug pairs. This has laid the foundation for the Clinical Pharmacogenomics Service becoming a billable service, and provides a potentially generalizable model.

## The future

One topic raised in multiple sessions that will be increasingly important in the future was the return of incidental findings, and this was the focus of Holly Tabor’s talk (University of Washington, USA). She provided an in-depth study of participant preferences regarding the return of incidental or secondary findings; 95% of patients in her sample said they would prefer unrestricted access to their incidental findings regardless of how applicable it was to their own known clinical condition. Interestingly, surveyed clinicians were more cautious in their recommendations for return of data.

The meeting concluded with an overview of the evolution of the technologies currently available and a preview of emerging technologies. It was evident both in the final session and throughout this meeting that the pace of the precision medicine field continues to quicken and, although questions remain, the findings from research are set to move from the bench to the bedside in the foreseeable future.

## Competing interests

The authors declare that they have no competing interests.

